# Behavioural Stress Responses Predict Environmental Perception in European Sea Bass (*Dicentrarchus labrax*)

**DOI:** 10.1371/journal.pone.0108800

**Published:** 2014-09-29

**Authors:** Sandie Millot, Marco Cerqueira, Maria-Filipa Castanheira, Øyvind Øverli, Rui F. Oliveira, Catarina I. M. Martins

**Affiliations:** 1 CCMAR-CIMAR L.A., Universidade do Algarve, Campus de Gambelas, Faro, Portugal; 2 Norwegian University of Life Sciences, Department of Animal and Aquacultural Sciences, Ås, Norway; 3 ISPA Unidade de Investigação em Eco-Etologia Integrative Behavioural Biology Group, Lisboa, Portugal; 4 Champalimaud Neuroscience Programme, Instituto Gulbenkian de Ciência, Oeiras, Portugal; Liverpool John Moores University, United Kingdom

## Abstract

Individual variation in the response to environmental challenges depends partly on innate reaction norms, partly on experience-based cognitive/emotional evaluations that individuals make of the situation. The goal of this study was to investigate whether pre-existing differences in behaviour predict the outcome of such assessment of environmental cues, using a conditioned place preference/avoidance (CPP/CPA) paradigm. A comparative vertebrate model (European sea bass, *Dicentrarchus labrax*) was used, and ninety juvenile individuals were initially screened for behavioural reactivity using a net restraining test. Thereafter each individual was tested in a choice tank using net chasing as aversive stimulus or exposure to familiar conspecifics as appetitive stimulus in the preferred or non preferred side respectively (called hereafter stimulation side). Locomotor behaviour (*i.e.* time spent, distance travelled and swimming speed in each tank side) of each individual was recorded and analysed with video software. The results showed that fish which were previously exposed to appetitive stimulus increased significantly the time spent on the stimulation side, while aversive stimulus led to a strong decrease in time spent on the stimulation side. Moreover, this study showed clearly that proactive fish were characterised by a stronger preference for the social stimulus and when placed in a putative aversive environment showed a lower physiological stress responses than reactive fish. In conclusion, this study showed for the first time in sea bass, that the CPP/CPA paradigm can be used to assess the valence (positive *vs.* negative) that fish attribute to different stimuli and that individual behavioural traits is predictive of how stimuli are perceived and thus of the magnitude of preference or avoidance behaviour.

## Introduction

How and for what reasons individuals differ in the way they react to potential risks, handle novelty, or interact with conspecifics remain fascinating questions. Scherer [1] suggested that the individual evaluates the significance of an event according to a set of stimulus evaluation checks. These evaluate the relevance of the event according to various dimensions (*e.g.* novelty, pleasantness, and importance of the event for the individual), its implication in terms of the individual's needs, the possibility for the individual to cope with the event and the compatibility of the event with social or individual standards. A variety of related concepts have been used to describe individual differences in behaviour that are consistent over time and across situations (see Budaev and Brown [2]). Wilson et al. [3] proposed that the shy-bold continuum (propensity to take risk) is a fundamental axis of behavioural variation in many species. Another concept frequently used in the study of animal personality is behavioural syndrome: a suite of correlated behaviours that are expressed either within a given context or across context [4]. A third concept frequently used to investigate individual differences in behaviour is coping styles or strategies. Two alternative coping styles are frequently distinguished: proactive and reactive [5]–[7]. Proactive individuals are more active, aggressive, bold, tend to form inflexible routines and learn more slowly about small changes in the environment. Reactive individuals, in contrast, are shyer, non-aggressive and more sensitive to environmental changes. The existence such contrasting phenotypes seems to be a widespread phenomenon, with some aspects of this individual variation being reported in invertebrates (e.g. squids, *Euprymna tasmanica* [8]), lizards (*Anolis carolinensis* [9]) and in various species of fish (sticklebacks, *Gasterosteus aculeatus* [10]–[12], rainbow trout, *Oncorhynchus mykiss*, [13]–[15]; Nile tilapia, *Oreochromis niloticus*, [16], [17]; Gilthead sea bream, *Sparus aurata*, [18], [19]). Far from being stereotyped and invariant, differences in the behavioural repertoires, learning and memory abilities observed in both phenotypes suggest that fish are curiously plastic [20].

Recent reviews of fish cognition suggest fish show a rich array of sophisticated behaviours. For example they have functional long-term memories, develop complex traditions, show signs of Machiavellian intelligence, cooperate with and recognise one another and are even capable of tool use [21]–[23]. Emerging evidences also suggest that, despite appearances, the fish brain is also more similar to higher vertebrate one than previously thought [24]–[26]. Although this amount of knowledge, the way fish perceive stimuli and the affective value they attribute to them are still poorly known phenomena.

For most people, this is either linked to animal sentience or consciousness. Sentience is quite difficult to define or measure, and the meaning is constantly debated by scientists and philosophers alike, but it might be summed up in an ethical context as the ability to experience pleasure and pain (*i.e.* subjective perceptual experiences [27], [28]). Being increasingly used in animal welfare evaluations and recognised as adaptive products of natural selection [29], affective states are not directly observable, and behavioural and physiological proxies have to be used in order to probe animal affective states. Preference/avoidance and motivation tests have been used for this purpose, based on the assumption that affective states are linked to motivation/preference and ultimately drive behaviour [30]. In these tests the animal is given some control over its environment, so that we can observe their choices in preference tests, or how much they are willing to work to access or avoid given resources or threats in operant motivation tests [30]–[33]. Thus, the ability of fish to express choice according to their preference or avoidance is a well established phenomenon; however, the extent of intraspecific variation and whether coping style influences this type of behaviour are still unknown.

In this study we investigated how European sea bass (*Dicentrarchus labrax*), one of the most important commercial species in Europe, evaluated putative appetitive (presence of social partners) or putative aversive (net chasing) stimuli in a conditioned place preference/avoidance test (CPP/CPA [34], [35]). CPP/CPA is a behavioural paradigm in which a reward or a punishment is paired repeatedly with environmental cues so that the animal associates the cues with the appetitive or aversive stimulus and eventually develops preference or avoidance for the marked location even in the absence of the stimulus [36]. In order to validate this paradigm as a gauge of valence attributed by the fish to the putative appetitive/aversive stimuli, we used physiological (cortisol and glucose) and behavioural (distance travelled and swimming speed) measures. In addition, we investigated whether pre-existing differences in the behavioural response to acute stress (a putative indicator of stress coping style or animal personality in fish [18], [37]) would predict individual variation in the response to putative appetitive and aversive stimuli.

## Materials and Methods

The experiment described was conducted in accordance with the Guidelines of the European Union Council (86/609/EU) and Portuguese legislation for the use of laboratory animals, and approved by the ethics committee from the Veterinary Medicines Directorate, the Portuguese competent authority for the protection of animals, Ministry of Agriculture, Rural Development and Fisheries, Portugal. Permit number 0420/000/000-n.99-09/11/2009. The rules and regulations which protect experimental animals from unnecessary pain and suffering have been strictly followed during the experiment. In preparing the experiment, we have carefully considered the application of the 3R (Replacement, Reduction and Refinement, 2010/63/UE).

### 1. Experimental fish, housing and feeding

Fish were hatched and reared at the experimental research station of Ifremer in Palavas-les- Flots (France) until they weighted 0.1 g and then transported to Ramalhete Research Station (Faro, Portugal). Fish were housed in stock tanks (500 L) under sea bass standard rearing conditions [38] during 8 months before the start of the experimental procedures (rearing density from 0.3 kg m^−3^ (mean fish weight = 0.1 g) to 10 kg m^−3^ (mean fish weight = 45 g) which are considered as low rearing densities and reach all welfare demand). Fish were fed a commercial diet (Aquagold 3 mm, Aquasoja, Sorgal SA, Portugal; 44% crude protein, 14% crude fat, 8% ash, 2.5% crude fibres, 1.0% phosphorus) using automatic feeders (1.5% BW day^−1^). Fish were reared in open water circuit tanks, with a temperature of 20±7°c, salinity of 35±2‰ and dissolved oxygen above 95%, and a 12L∶12D photoperiod was employed with light on at 08:00. One month before the start of experimental procedures, 90 fish were randomly selected, anaesthetised with 2-phenoxyethanol (0.3‰, Sigma-Aldrich) and individually identified with a PIT-tag (Micro BE, France) injected in the flesh under the dorsal fin and with a visible implant elastomer tag (VIE; Northwest Marine Technology, USA) in the caudal fin. At the start of the experiment the body mass of the fish was 45±1.3 g (mean ± SE).

### 2. Set up and experimental procedures

#### 2.1. Restraining test

Escape behaviour during restraining or confinement has been used to discriminate personality traits as well as physiological correlates of coping style in different fish species [16], [37], [39], [40]. More recently, Castanheira et al. [18] showed in sea bream that escape behaviour during restraining was consistent over time and across contexts. Moreover, Ferrari S., Millot S., Leguay D., Chatain B., Bégout ML (unpubl. data) demonstrated in sea bass that escape attempts during restraining test were negatively correlated to plasma cortisol concentration. For these reasons, the net restraining test was performed only one time 15 days before the conditioned place preference/avoidance (CPP/CPA) tests. The restraining test consisted of holding each fish individually in an emerged net for three minutes [16]–[19], [39], [41]. The following behaviours were measured: latency to escape (time in seconds taken by each fish to show an escape attempt; escape attempt was defined as a elevation of the body from the net); number of escape attempts and total time spent on escape attempts (total time in seconds taken by each fish escaping since the first to the last escape attempts). Behaviours measured were collapsed into first principal component scores using Principal Components Analysis (PCA) in order to obtain a score allowing the characterisation of coping styles. PC1 explained 86% of the variation and the number of escapes was the variable that contributed the most for PC1 (Table 1).

**Table 1:** Mean ± SEM, minimum (Min.) and maximum (Max.) values of behavioural variables obtained for the restraining test (N = 90) and PCA loading used to generate a principal component scores (PC1).

Behavioural variables	Mean ± SEM	Min.	Max.	Loadings for PC1	Eigenvalues	% variation explained
Latency escape (s)	90.9±0.75	2	180	−0.855	85.991	85.991
Number escape	5.3±0.06	0	18	0.962	12.481	
Total escape time (s)	1.6±0.02	0	6.8	0.96	1.528	

Fish presenting a high latency to escape, small number of escape attempts and shorter total time to escape were characterised by a low score and identified as reactive fish. On contrary, fish presenting a lower latency to escape, high number of escape attempts and longer total time to escape were characterised by a high score and identified as proactive (based on Silva et al. [39]; Martins et al. [16], [17] and Castanheira et al. [18], [19]). No threshold was applied to separate subjectively the fish in two categories *i.e.* proactive and reactive. Instead these data (Coping Style, CS, score) were used as a continuous variable.

#### 2.2. Conditioned place preference/avoidance test

Four days before the start of the CPP/CPA test, 3 groups of 12 fish each (6 focal fish with distinct VIE (Visible Implant Elastomer tag) and 6 familiar conspecifics, *i.e.* all fish coming from the same rearing tank) were placed in 3 different 100 L home tanks located in the experimental room. This was done to acclimatize fish to their new environment. The photoperiod and the water temperature, salinity and oxygenation were the same as in rearing tanks. The fish were fed *ad libitum* each morning. This procedure was repeated 5 consecutive times in order to test 30 fish per treatment: appetitive (APP), aversive (AVER) and control (CONT).

**Figure 1:**
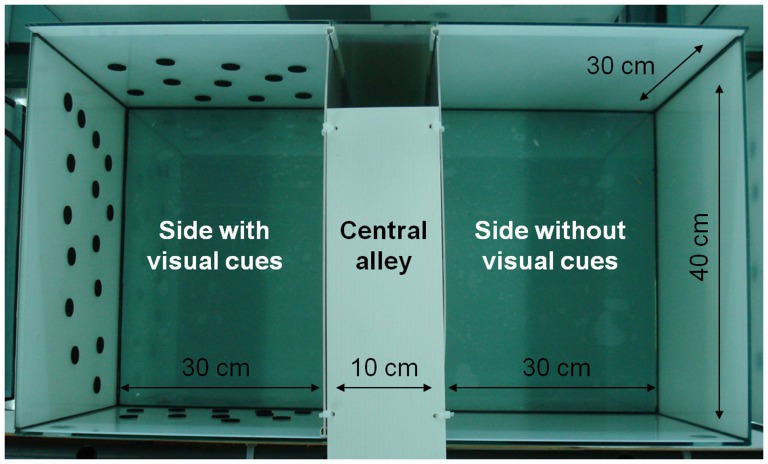
Experimental tank. CPP/CPA test glass aquarium (80L) divided into three compartments: one central alley with grey walls and two lateral compartments with white walls and with or without visual cues (black dots).

The CPP/CPA test was performed in 6 glass aquaria of 80 L (70 cm length×40 cm width and 30 cm depth). Each aquarium was divided into two halves by a 10 cm wide grey central alley: one half marked by white walls without dots and the other half marked by white walls with black dots used as visual cues for fish (Figure 1). One infrared LED projector (IR-294S/60, Monacor) was placed beneath each aquarium.

The CPP/CPA test consisted of three experimental phases conducted over a 3 days period. An initial habituation phase was performed on the first day. During this phase, each fish was placed individually in the tank and allowed to swim freely throughout the whole tank for 40 min (this period was determined based on preliminary observations). For each individual the preference for white or dotted side (>50% time spent) was assessed through a 20 min additional recording. Then the fish was put back in the home tank. Animals that showed a very strong initial preference (>90% time spent) in either side or strong freezing behaviour (<500 cm distance moved) were excluded from the study because their initial position during the habituation phase could not be representative of their real preference for that side. Therefore, animals that showed an initial preference between 50.1% and 89.9% for either side and which had moved more than 500 cm were used for data analyses. The habituation phase was followed by a conditioning phase, during which treatments differed between the appetitive and aversive stimulus groups. For the aversive stimulus (AVER), fish was placed in the same aquarium as during the habituation phase but had only access to the initial preferred side for 20 min, hereafter termed the stimulation side (SS). Afterwards, the fish was chased with a net during 10 s each 4 min for a period of 20 min. For the appetitive stimulus (APP), fish was placed in the non initial preferred side (new SS) for 20 min and then 2 familiar conspecifics were added in the tank for a period of 20 min. The control fish (CONT) were handled exactly the same way as the tested animals (maintained in the preferred side or in the non preferred side) except that the stimulus was omitted during the training phase. After each treatment, fish was placed back in the home tank.

The test phase was performed on the last day of the experiment (the third day) and consisted exactly of the same procedure as the habituation phase in order to record any behavioural changes.

After this last phase, fish were immediately caught and euthanized with an overdose of 2-phenoxyethanol (1‰, Sigma-Aldrich). Blood was thus sampled one hour after fish were transferred to the experimental aquarium (based on Fanouraki et al. [42]). Blood was withdrawn within 3 min from the caudal vein using heparinised syringes and centrifuges at 2000× g for 25 minutes at room temperature. After centrifugation plasma was frozen in dry ice and stored at −80°c for glucose (QCA, Spain) kit analysis. Plasma cortisol levels were measured by means of a commercial ELISA kit RE52061 (IBL Hamburg, Germany), with a sensitivity of 2.5 ng/ml, and intra and inter-assay coefficients of variation (CV) of 2.9 and 3.5%, respectively. After blood sampling, fish were identified and measured for standard length (cm).

During each phase, individual behaviour was recorded by infrared sensitive video camera (TVCCD-623-COL, Monacor, Denmark) equipped with infrared filter (dark red, Schneider Optics, USA) and positioned 1 m above the tank. The videos were stored in AVI files on a hard drive and analysed afterwards with the Lolitrack 2.0 software (Loligo Systems, Denmark). Before each video analyses, the background image of each tank was divided into three arenas (Arena 1 = white side, Arena 2 = grey middle alley, Arena 3 = dotted side). For each tank the background was calibrated by marking the length of the Arena 2 in the image and entering its actual value (10 cm). The Lolitrack 2.0 software tracks the fish as a dark object on a light background. By using infrared light underneath the tank we avoid light reflexion on the water surface and optimise the fish tracking by the software. The following parameters were quantified by the software: time spent in each arena (min), distance travelled in each arena (m) and the swimming speed in each arena (cms^−1^). In order to remove the influence of fish size in swimming speed data, these values were transformed in body length per second (BLs^−1^). To evaluate the fish behavioural changes between the habituation and the test phase, percent change of time spent, distance travelled and swimming speed were calculated as: [(Test phase value – Habituation phase value)/Habituation phase value]×100.

### 3. Statistics

Behaviours measured in restraining test were collapsed into first principal component scores (PC1) with orthogonal rotation (varimax) using Principal Components Analysis (PCA). The correlation matrix was used to check for multicollinearity (*i.e.* to identify variables that did not correlate with any other variable, or correlate very highly, r = 0.9, with one or more variables). Kaiser–Meyer–Olkin (KMO) test for sample adequacy was always greater than 0.5 and the Bartlett's test of sphericity was significant for all tests, indicated that correlations between items were sufficiently large for PCA. PCA analyses were performed using SPSS 18.0 for windows. The results are expressed as mean ± standard error of the mean (SEM).

All other statistical analyses were performed using Statistica 7 software (Statsoft, USA). The results were expressed as mean ± standard error of the mean (SEM).

A null model of side preference was tested by comparing the observed fish distribution to the theoretical homogeneous distribution in the side with or without dots (50% in each side) by a Kolmogorov–Smirnov test. Fish did not show a systematic initial preference for one side or the other one. Consequently, the stimulation was performed 36 times in the side without dots *vs* 42 times in the side with dots (d = 0.28; p>0.05).

One way ANOVA was used to analyse the differences in percent change of time spent on SS (after arcsine(sqrt (x/100)) transformation) by the experimental (APP or AVER) vs. control fish. Repeated-measures ANOVA's were used to analyse the differences in distance travelled (m) and swimming speed (BLs^−1^) between experimental and control fish, experimental phases (*i.e.* before and after conditioning phase) and tank sides (4-levels repeated factor: before SS *vs.* before non-stimulation side (nSS) *vs.* after SS *vs.* after nSS; categorical variables: experimental *vs.* control fish). Newman & Keuls tests were subsequently used to test differences between the habituation and the test phase of each treatment and between the control and the experimental groups both at the habituation and at the test phase of each treatment.

One way ANOVA followed by Newman & Keuls tests were used to analyse the differences in plasma concentrations of cortisol (ngml^−1^) and glucose (mmol^−1^) between experimental and control fish.

For both APP and AVER treatments, Pearson correlations matrices between time spent on SS, distance moved in SS and nSS, percent change of time spent on SS, percent change of distance moved in SS and nSS, plasma concentration of glucose and cortisol with Coping Style (CS) score were calculated. The significance level of each correlation matrice was defined according to the table of critical values of Pearson correlation coefficient corrected by the individual number (n) in Scherrer p792 [43].

## Results

From the 90 fish tested in this study, 12 fish did not comply with our CPP acceptance criteria and were thus removed from the analysis. This resulted in the following sample sizes: n = 28 for APP, n = 23 for AVER and n = 27 for CONT.

### 1. Restraining test

During the restraining test, fish waited on average 91 s before the first escape attempt and they performed a mean of 5 escape attempts for a total escape time of around 2 s (Table 1).

### 2. Time spent on the stimulation side

**Figure 2:**
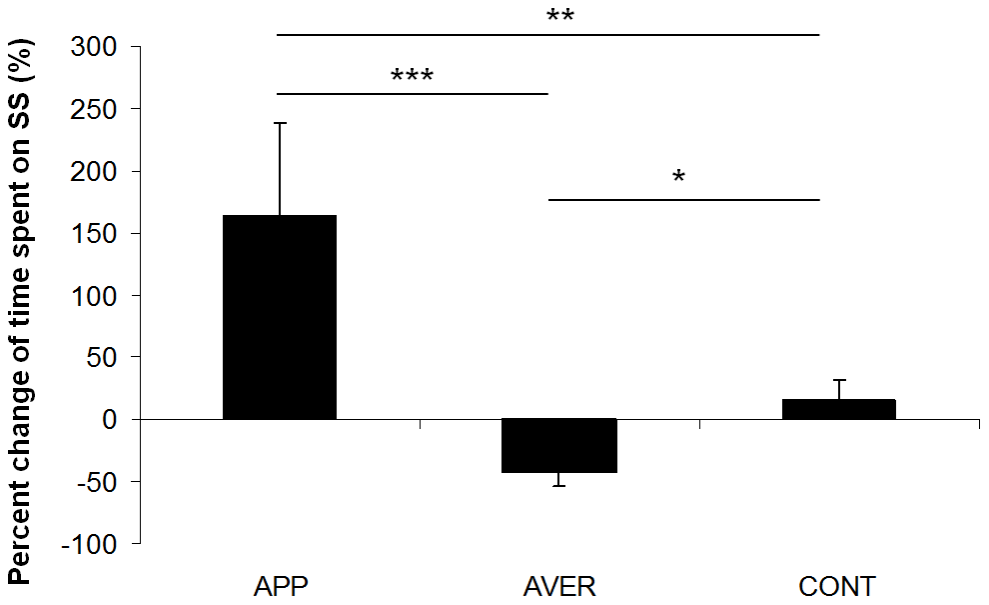
Time spent on the stimulation side. Percent change of time spent (Mean ± SEM; in %) by the fish on the stimulation side between the habituation and test phases for each treatment. One way ANOVA, * <0.05; ** <0.01; *** <0.001.

On average fish subjected to the appetitive stimulus showed a high increase (+163%) in the time spent on the SS during the test phase, whereas fish exposed to the aversive stimulus showed a significant decrease (−42%) in the time spent on the SS during the test phase (Fig. 2; One way ANOVA F_2,75_ = 6.60, p<0.01).

### 3. Distance travelled

**Figure 3:**
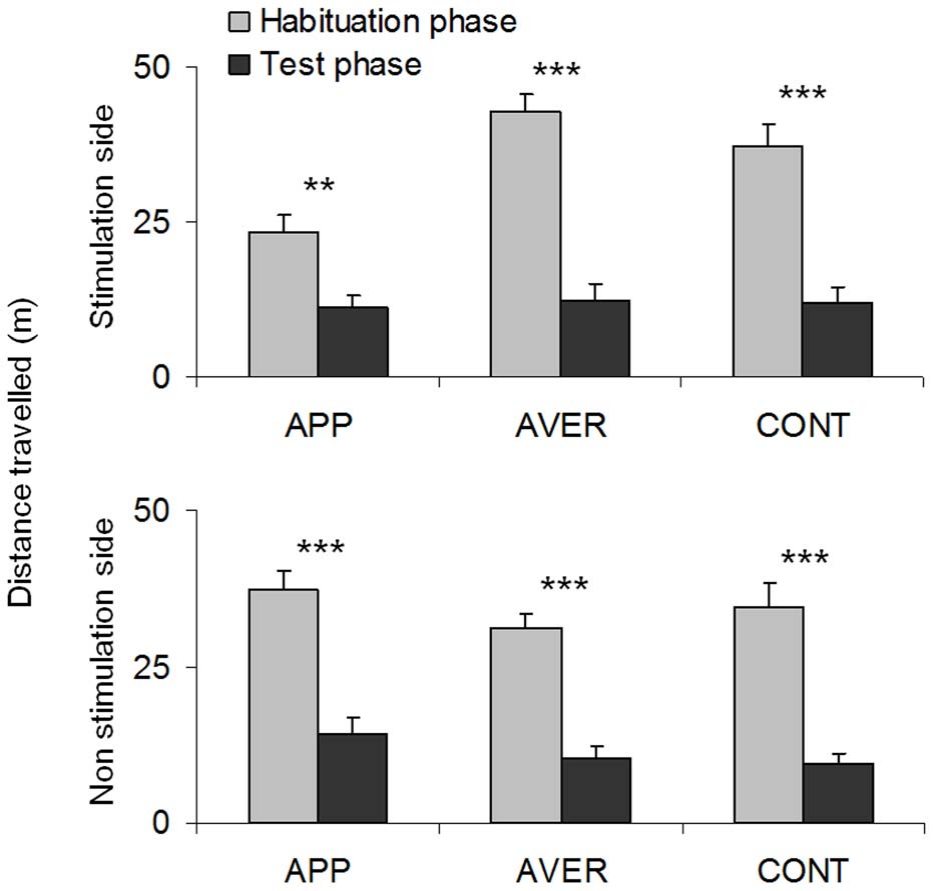
Distance travelled. Distance travelled (Mean ± SEM; in m) by the fish on the stimulation side and on the non stimulation side during the habituation and test phases for each treatment. Repeated ANOVA, ** <0.01; *** <0.001.

The main effect of treatment (*i.e.* APP *vs.* AVER *vs.* CONT groups) on the distance travelled was not significant (F_2,75_ = 0.54, p = 0.58), but there were a significant main effect of the repeated measure (*i.e.* before SS *vs.* before nSS *vs.* after SS *vs.* after nSS; F_3,225_ = 103.11, p<0.001) and a significant interaction between treatment and the repeated measure (F_3,225_ = 7.32, p<0.001). Thus, regardless of treatment or tank side, fish significantly decreased the distance travelled between the habituation and the test phase (Fig. 3).

### 4. Swimming speed

The main effect of treatment (*i.e.* APP *vs.* AVER *vs.* CONT groups) on swimming speed was not significant (F_2,75_ = 0.10, p = 0.90), but there were a significant main effect of the repeated measure (*i.e.* before SS *vs.* before nSS *vs.* after SS *vs.* after nSS; F_3,225_ = 50.47, p<0.001) and a significant interaction between treatment and the repeated measure (F_3,225_ = 2.94, p<0.01).

Whatever the treatment and the tank side, fish significantly decrease the swimming speed during the test phase (data not shown).

### 5. Blood plasma analysis

There was no significant difference between APP, AVER and CONT in plasma concentration of cortisol (237±24; 212±25; 270±20 ng ml^−1^ respectively; F_2,71_ = 1.11, p = 0.33) and glucose (4.32±0.1; 4.33±0.1; 4.26±0.1 mmol l^−1^ respectively; F_2,67_ = 0.14, p = 0.86).

### 6. Correlations between coping style, behaviour and physiology parameters

**Figure 4:**
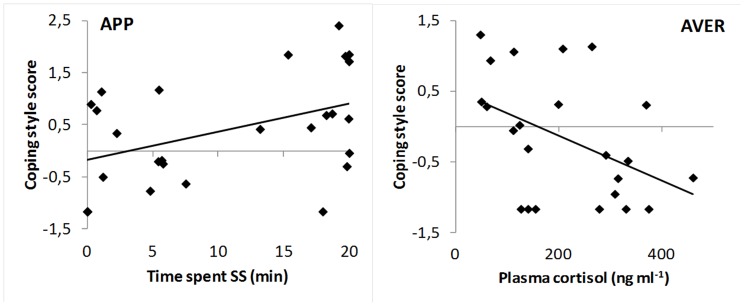
Correlations between coping style, behaviour and physiology parameters. Correlations between coping style score and time spent on SS (min) for appetitive treatment and coping style score and plasma cortisol concentration (ng ml^−1^) for aversive treatment. The full black lines represent the linear regressions.

The correlations matrice for the APP treatment showed that fish that spent the most time on SS during the test phase also had a higher CS score (Table 2, Fig. 4).

**Table 2:** Pearson correlations matrice and p value for appetitive (APP) and aversive (AVER) treatments between time spent on stimulation side (SS), distance moved in SS and on non stimulation side (nSS), percent change of time spent on SS, percent change of distance moved on SS and nSS and plasma concentration of cortisol and glucose with Coping Style score.

	APP		AVER	
	Coping style score		Coping style score	
Time spent SS (min)	**0.470**	**P = 0.019**	−0.225	P = 0.340
Distance moved SS (m)	−0.050	P = 0.796	−0.379	P = 0.099
Distance moved nSS (m)	−0.180	P = 0.384	−0.178	P = 0.452
Percent change time spent SS (%)	0.210	P = 0.030	−0.161	P = 0.498
Percent change distance moved SS (%)	−0.130	P = 0.539	**−0.441**	**P = 0.052**
Percent change distance moved nSS (%)	−0.240	P = 0.259	−0.169	P = 0.476
Cortisol (ng ml^−1^)	0.043	P = 0.841	**−0.495**	**P = 0.027**
Glucose (mmol l^−1^)	0.121	P = 0.573	0.009	P = 0.969

The significance level of each correlation matrice was defined according to the table of critical values of Pearson correlation coefficient corrected by the individual number (n) in Scherrer p792 [43] (APP: n = 28, |R|>0.374; AVER: n = 23, |R|>0.413). The significant results are in bold.

The correlations matrice for the AVER treatment showed that fish characterised by a low CS score increased the distance moved in SS and had higher plasma cortisol concentrations (Table 2, Fig. 4).

## Discussion

This study demonstrated that a single exposure to an appetitive or an aversive stimulus significantly increased or decreased respectively, the time spent by sea bass on the stimulation side of a CPP/CPA setup. This behavioural change clearly suggests that the experimental fish attributed a positive valence to the presence of social partners, and a negative valence to net chasing. Further, it would appear that altered place preference observed during the final test phase (in the absence of stimulus) is due to associative learning of visual cues (black dots) coupled to expected appetitive or aversive stimulus.

Notably, both experimental and control fish showed a strong decrease in swimming activity (distance travelled and swimming speed) in both sides of the tank. This result could be explained either by the fact that fish became habituated to the aquarium and thus reduced exploration or by the experimental procedure which consisted of handling fish each day to transfer them from their home tank to the experimental tank and which could have induced stress (*i.e.* freezing behaviour). Thus, even if fish showed preference or avoidance for the tank zone associated with appetitive or aversive stimulus respectively, they did not express a swimming activity comparable to that observed during the habituation phase. This last interpretation is supported by the similar high plasma concentrations of cortisol and glucose observed across treatments. Thus, the stimulus effect on fish physiology was probably masked by the stress due to the handling procedure. To summarise, even if the experimental set up to assess the affective value that sea bass attribute to a stimulus was not optimal due to the handling procedure which has masked part of the behavioural and physiological responses, the results showed nevertheless that fish can learn, remember and make decisions to avoid being exposed to aversive stimulus (net chase) or in contrary to seek for appetitive stimulus (familiar conspecifics).

The consistency of behaviours has not been tested in this study and should be done in the future in order to clearly define if the behavioural stress response measured during the restraining test could be considered as coping style, behavioural trait or personality. However, restraining test has been shown to be repeatable in sea bream [18] and correlated with physiological stress in sea bass (see method section) and thus the observed behaviour is likely to be a coping style. More interestingly, this study showed that behavioural stress response or coping style modulates the response (*i.e.* appraisal) of appetitive and aversive stimuli. When fish were subjected to an appetitive stimulus, proactive individuals expressed a higher preference (*e.g.* time spent) for the stimulation side than reactive fish. But when fish were submitted to an aversive treatment, reactive fish exhibited an increase of distance moved in the stimulation side (anxiety) and a higher plasma cortisol concentration than proactive fish. These behavioural and physiological changes showed that fish exhibiting proactive behaviour were more responsive to the appetitive stimulus while reactive phenotypes responded to the aversive one. These results suggested also that the proactive phenotype is less fearful when presented with a signal previously associated with an aversive stimulus, as compared to individuals of the reactive type. Previous studies have demonstrated in fish relationship between anxiety or fear behaviour and corticosterone response [44] and limbic neural systems [7], [29] and also between fearfulness and coping style [16], but it is the first time that a study highlights the link between behavioural stress and fear responses and physiological patterns simultaneously.

Even if correspondence between boldness and sociability is not clearly established (Cote et al., 2010; Trompf and Brown, 2014), in our study, proactive fish seemed more responsive to social stimulus than the reactive ones. Pike et al. [45] showed in three-spined stickleback (*Gasterosteus aculeatus*) that bolder individuals had fewer overall interactions than shy fish, but tended to distribute their interactions more evenly across all group members. Thus, the fact that proactive fish were more attracted by the side where congeners were present did not necessarily means that they are more social but simply that they attribute a higher positive value to this stimulus than reactive fish.

In conclusion, this study showed for the first time in sea bass, that the CPP/CPA paradigm can be used (with some set up improvements in order to reduce the fish freeze behaviour due to the handling procedure) to assess the valence (positive *vs.* negative) that fish attribute to environmental stimuli and that the individual's behaviour under stress predicts how stimuli are perceived and thus the subsequent preference or avoidance behaviour.

## Supporting Information

File S1 Supporting information file shows data of restraining test, CPP-CPA test and blood analysis.
(XLSX)
